# Detection of basal cell carcinoma on whole-slide images from Mohs micrographic surgery using weakly supervised learning

**DOI:** 10.1016/j.jdin.2026.07.004

**Published:** 2026-07-20

**Authors:** Kajsa Villiamsson, Ludvig Fornstedt, Geert Litjens, Avital L. Amir, Nelli Sjöblom, Anna Kaatonen, Olivia Vesala, Filmon Yacob, John Paoli, Noora Neittaanmäki

**Affiliations:** a Department of Laboratory Medicine, Institute of Biomedicine, Sahlgrenska Academy, University of Gothenburg, Gothenburg, Sweden; b Department of Clinical Pathology, Sahlgrenska University Hospital, Gothenburg, Region Västra Götaland, Sweden; c Department of Physics, Chalmers University of Technology, Gothenburg, Sweden; d Department of Pathology, Research Institute for Medical Innovation and Oncode Institute, Radboud University Medical Center, Nijmegen, The Netherlands; e Department of Pathology, Radboud University Medical Center, Nijmegen, The Netherlands; f Department of Dermatology and Allergology, University of Helsinki and Helsinki University Hospital, Helsinki, Finland; g Ekkono Solutions, Varberg, Sweden; h Department of Dermatology, Institute of Clinical Sciences, Sahlgrenska Academy, University of Gothenburg, Gothenburg, Sweden; i Department of Dermatology and Venereology, Sahlgrenska University Hospital, Gothenburg, Region Västra Götaland, Sweden; j Department of Dermatology and Venereology, Institute of Clinical Medicine, School of Medicine, Faculty of Heath Sciences, University of Eastern Finland, Kuopio, Finland

**Keywords:** artificial Intelligence, basal cell carcinoma, deep learning, foundation models, Mohs micrographic surgery, tumor detection, vision models, weakly supervised

## Abstract

**Background:**

Mohs micrographic surgery (MMS) is the gold standard for treating aggressive basal cell carcinoma, but its success depends on expertise in intraoperative interpretation of frozen sections.

**Objective:**

To develop weakly supervised, multiple instance learning framework using a pathology foundation model for automated basal cell carcinoma detection in MMS frozen sections.

**Methods:**

An internal data set of 995 frozen MMS whole-slide images was slide-level labeled as tumor (512) or no tumor (483). Furthermore, tumor regions in the test set were annotated. Whole-slide images were tiled and encoded with Prov-Gigapath features for a weakly supervised multiple instance learning framework. The performance was evaluated as binary slide-level classification and in attention maps showing the localization of the tumor regions prior to validation on 2 external data sets.

**Results:**

The model showed near-perfect diagnostic performance, achieving 97.0% accuracy, and an area under the receiver operating characteristic curve of 0.998 on the internal data set. Furthermore, attention maps visualized diagnostically relevant regions, enhancing model interpretability (Intersection-over-Union = 0.41 ± 0.046 [Dice 0.58 ± 0.046]). External validation confirmed robust performance (90% to 92% accuracy, area under the receiver operating characteristic curve: 0.92-0.95).

**Limitations:**

The absence of tumor region annotations on external data sets.

**Conclusion:**

These findings support the feasibility of artificial intelligence–assisted analysis of MMS frozen sections and justify prospective studies evaluating its integration into clinical workflows.

## Graphical abstract

**Figure:**
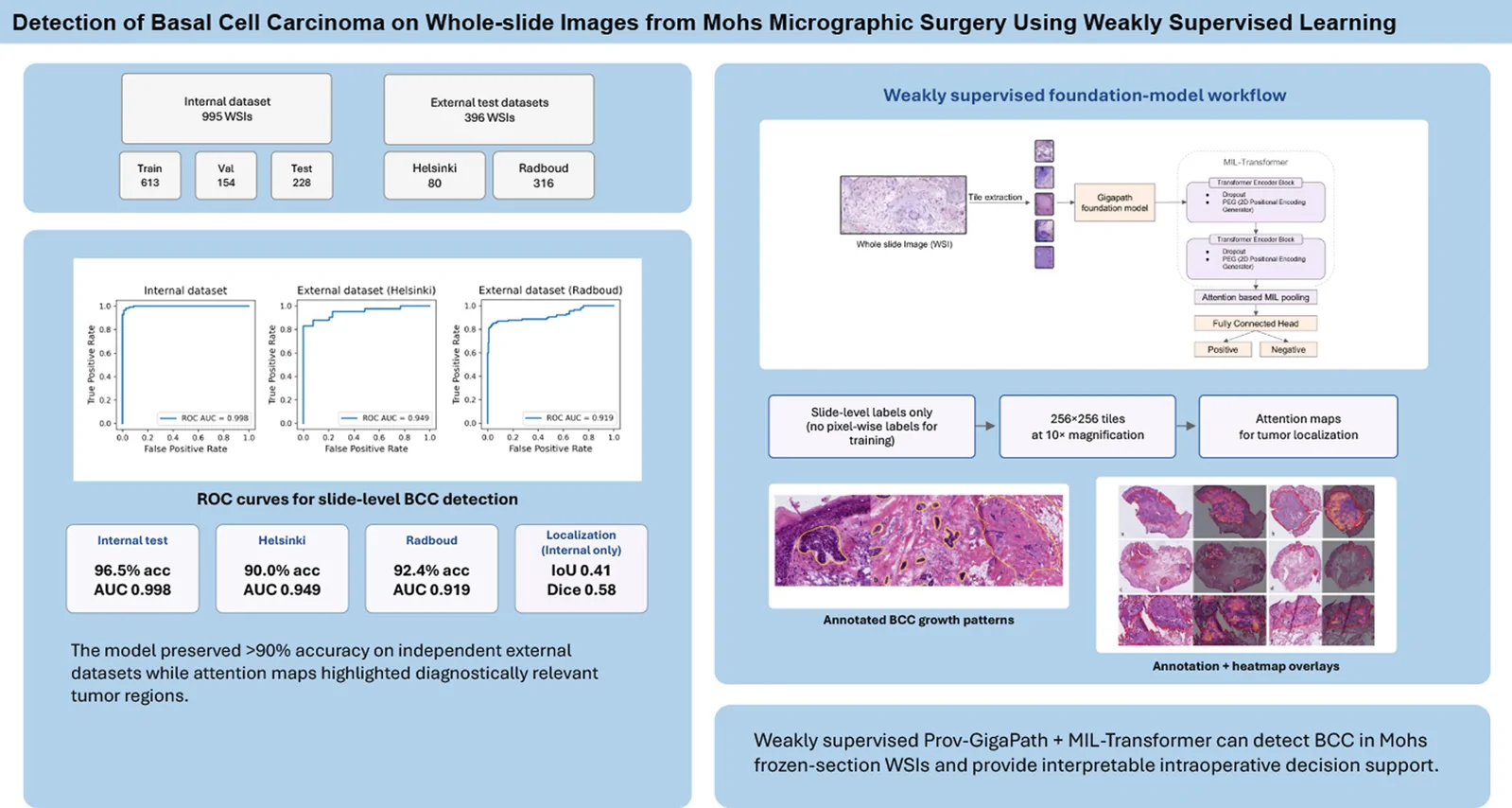

**Capsule Summary**

• This study advances Mohs micrographic surgery by introducing a weakly supervised multiple instance learning framework for tumor detection in frozen sections, achieving 97.0% accuracy and 0.998 area under the receiver operating characteristic curve.
• The approach demonstrates the potential for automated basal cell carcinoma detection in Mohs micrographic surgery and warrants further evaluation in prospective clinical settings.

## Background

Basal cell carcinoma (BCC) is the most common type of skin cancer, and the incidence is rising.^1^ BCC very rarely metastasizes, but it can grow aggressively locally and destroy important anatomical structures causing significant morbidity. An accurate diagnosis is crucial for successful treatment.^2^ The current diagnostic gold standard is histopathological evaluation, in which BCCs are categorized as low-risk or high-risk subtypes. High-risk subtypes typically exhibit more aggressive growth patterns and carry a greater risk of treatment failure or recurrence.^3^

For high-risk BCC subtypes, especially in cosmetically and functionally critical facial areas, Mohs micrographic surgery (MMS) remains the gold standard. Internationally, MMS offers the lowest recurrence rates by combining stepwise histopathological assessment of frozen sections with complete intraoperative margin control while preserving healthy tissue.^4^ However, the success of MMS is dependent upon the physician’s ability to interpret intraoperative frozen sections and correctly identify the residual tumor, which can be challenging and requires extensive training. The discordance between certified MMS surgeons and dermatopathologists interpreting MMS slides has been shown to vary from 0.2% to 6.3%.^5^^,^^6^ In challenging cases, an additional review of MMS slides by a dermatopathologist may be needed.^6^ Automation could support slide interpretation by the Mohs surgeon or provide adjunctive intraoperative decision support.

Digitalization has enabled the use of computational pathology tools. Numerous studies in computational pathology rely on pixel-wise annotations with specific regions needing to be delineated on whole-slide images (WSIs). This is very time-consuming, especially considering that very large amounts of data are required in training deep learning algorithms from scratch for them to perform well.^7^ In contrast, weakly supervised learning approaches, particularly multiple instance learning (MIL), relies solely on slide-level labels and requires no pixel-wise annotations making large-scale computational pathology studies more feasible.^8^

The recent development of self-supervised learning algorithms has enabled training deep neural networks on large unlabeled data sets, yielding results on par with supervised learning strategies without the need for laborious training annotations.^8^, ^9^, ^10^, ^11^, ^12^ Building on this paradigm, foundation models, large deep neural networks trained on large clinical data, can now be adapted for a variety of downstream tasks with minimal to no fine-tuning.^13^, ^14^, ^15^, ^16^, ^17^ A number of pathology-specific foundation models have been made publicly available.^18^ The ability to use foundation models as a base and fine-tune them for new tasks has lowered barriers for smaller research groups to participate in computational pathology research and accelerated the translation of artificial intelligence (AI)–based methods into clinical practice.^19^^,^^20^

In this direction, several studies have successfully applied supervised and/or weakly supervised deep learning algorithms to detect and classify BCCs on routinely processed paraffin-embedded sections.^18^^,^^21^ In contrast, relatively few studies have focused on frozen MMS sections. In MMS, it is crucial to correctly locate the tumor regions to avoid recurrences and minimize the number of surgical stages. Thus, more accurate algorithms for this task are warranted.

In this study, we aimed to develop a weakly supervised, MIL framework using a pathology foundation model for automated BCC detection in MMS frozen sections.

## Methods

### Internal data set

The inclusion criteria comprised BCCs excised during MMS surgery at the Department of Dermatology, Sahlgrenska University Hospital, between 2018 and 2023. Cases were included if at least 1 representative hematoxylin and eosin–stained frozen-section glass slide was available for digitization during the data collection period (2023-2024). All slides were reviewed to confirm adequate preservation of tissue morphology and hematoxylin and eosin staining quality. However, cases with common frozen-section artifacts, including tissue folds, freezing artifacts, minor fragmentation, and staining variability, were not excluded, as these reflect routine clinical practice and contribute to the real-world variability of the data set. A single representative slide per case was selected including different stages of MMS. The negative cases were derived from routine MMS surgery specimens and were not selected to exclude common mimickers or artifacts. Consequently, the negative class included slides containing features such as inflammation, scar tissue, adnexal structures, basaloid proliferations, and typical frozen-section artifacts. The glass slides were anonymized and subsequently scanned digitally using whole-slide imaging in 40x mode (0.23 μm/pixel, 20x objective lens) using the Nanozoomer S210 Digital Slide Scanner (Hamamatsu Photonics K.K.). The internal data set was split into training, validation, and test sets, as shown in Table I.

**Table I:** Training, validation, and test data ratio of internal data set

	BCC positive	BCC negative	Total
Internal data set			
Training set	320 (52%)	293 (48%)	613
Validation set	80 (52%)	74 (48%)	154
Test set	113 (50%)	115 (50%)	228
Total	513 (52%)	482 (48%)	995
External data set; Radboud University medical center	114 (36%)	202 (64%)	316
External data set; Helsinki University Hospital	41 (51%)	39 (49%)	80

Data ratio of external data sets.

*BCC*, Basal cell carcinoma.

### Data labeling

**Fig 1:**
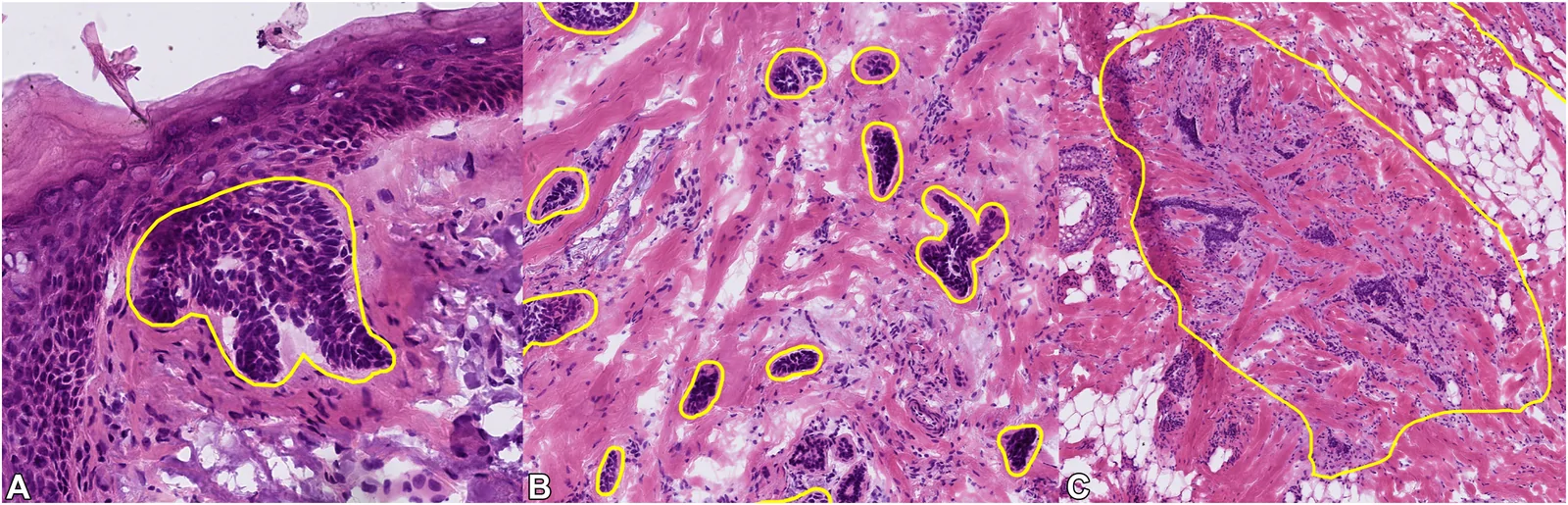
Examples of BCC growth patterns present in the internal test set frozen MMS sections, with yellow marking of areas annotated as containing BCC. **A,** Superficial growth pattern, **(B)** micronodular growth pattern, and **(C)** infiltrative growth pattern. In cases where the tumor was represented as very narrow tumor cords, the surrounding stroma was also annotated. Hematoxylin and eosin stain. *BCC*, Basal cell carcinoma; *MMS*, Mohs micrographic surgery.

The 995 WSIs in the internal data set were annotated on a slide level by a resident in Pathology and a Dermatopathologist as ‘BCC positive’ or ‘BCC negative’ based on the presence or absence of tumor. Furthermore, the WSIs (*n* = 113) containing a tumor in the test set were annotated pixel-wise by drawing the tumor regions on the slides using the open-source platform Quantitative Pathology & Bioimage Analysis.^22^ In the cases where the 2 main annotators had differing opinions, a third annotator (a senior dermatopathologist) was brought in to achieve consensus on a final annotation decision. Fig 1 shows examples of annotated tumor slides.

### External data sets

The external data were collected from 2 medical centers. A total of 316 WSIs, between 2016 and 2021, were scanned during 2023 at Radboud University Medical Center (Nijmegen) with a 3DHistech Pannoramic 1000 (3DHistech) digital slide scanner at 40x magnification with a pixel size of 0.24 mm. An additional 80 WSIs between 2023 and 2025 were scanned at the Department of Dermatology, Helsinki University Hospital (Helsinki) with a Leica Aperio GT 450 (Leica Biosystems) scanner. These slides were also labeled on the slide level as ‘BCC positive’ or ‘BCC negative’ (Table I). For the external data sets, we applied the same criteria as for the internal data set: 1 representative slide per case across varying MMS stages. Negative cases were drawn from routine MMS specimens without excluding mimickers or artifacts.

### Model architecture and training

The WSIs were tiled with the open-source software DeepZoom (OpenSlide.org) into fixed-size, square patches at 10× magnification and with a size of 256 × 256 pixels. From the tiles, tile-level feature vectors were encoded with a pathology foundation model using the Gigapath inference pipeline.

**Fig 2:**
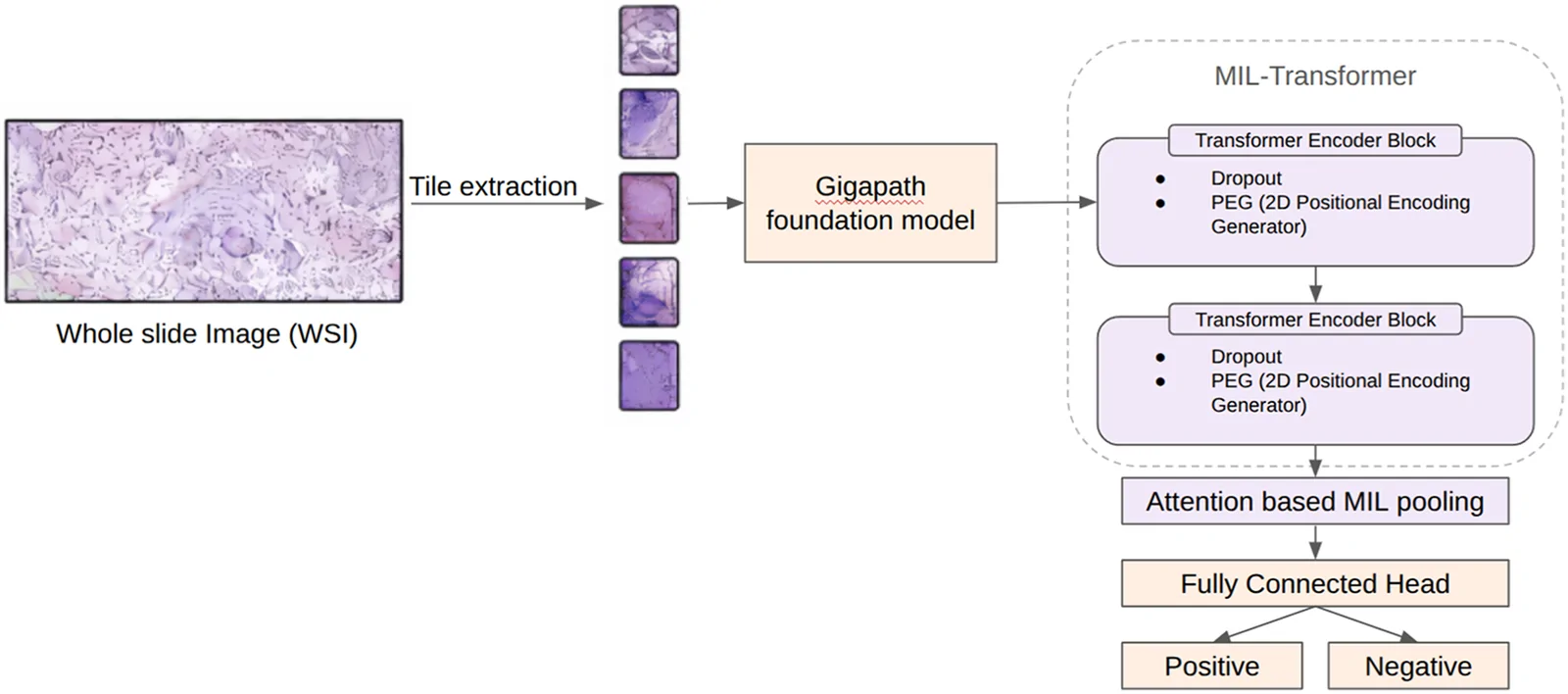
Model architecture.

The model architecture is shown in Fig 2. We adopted a weakly supervised MIL framework that operates on tile-level feature embeddings extracted from WSIs, with slide-level labels indicating the presence or absence of tumor. For each slide, the tile embeddings were processed jointly with their corresponding spatial coordinates. An MIL-Transformer architecture was employed, in which tile features were first projected into a 256-dimensional latent space and then passed through 2 Transformer encoder blocks with 4 attention heads and a dropout rate of 0.2. To explicitly model spatial context, each encoder block incorporated a 2-dimensional positional encoding generator, implemented as a depth-wise convolution over a reconstructed tile grid, enabling learnable, content-adaptive positional encoding based on the spatial arrangement of tile embedding.^23^

Following spatially enriched self-attention, tile representations were aggregated into a slide-level embedding using an attention-based pooling mechanism, and final classification was performed with a fully connected prediction head. Model training was conducted using cross-entropy loss with label smoothing and the AdamW optimizer. Mixed-precision training and gradient norm clipping were employed, together with a learning-rate schedule consisting of a short linear warm-up followed by cosine annealing.

### Model evaluation

For model evaluation, the optimal checkpoint was loaded into the MIL-Transformer architecture and applied to a separate, held-out test set of slides to obtain the predicted labels. We summarized the model’s performance using slide-level metrics, including accuracy, true/false positives and negatives (true positive, true negative, false positive, and false negative), and area under the receiver operating characteristic curve (AUC).

To assess qualitative localization, we generated heatmaps for the true-positive cases. This was achieved by using the attention scores of each instance embedding (patch) as an indicator of regions the model focused on. The attention scores from the pooling module were minimum to maximum normalized and subsequently projected back onto the original tile grid.

Furthermore, we quantified the generated heatmaps using Intersection-over-Union (IoU). Activated heatmap regions were thresholded and converted into rectangular polygons corresponding to patch locations, while annotations were represented as vector polygons parsed from GeoJSON files and scaled to a common thumbnail coordinate system. Both geometries were restricted to tissue cores containing annotations and activated patches, merged separately using polygon union operations, and subsequently combined to compute the IoU as the ratio between the area of intersection and the area of union. All geometric operations (union, intersection, and area computation) were performed using Shapely.

### Validating model on external data sets

Model selection was based on a held-out validation split processed identically to training but without augmentations. After each epoch, we computed validation cross-entropy and accuracy by forwarding complete slide bags through the model done on 2 independent data sets (Radboud University Medical Center and Helsinki University Hospital) using the identical inference pipeline. The external data sets were used only for final validation and not for model selection, threshold tuning, or training.

## Results

### Internal testing on slide level

**Fig 3:**
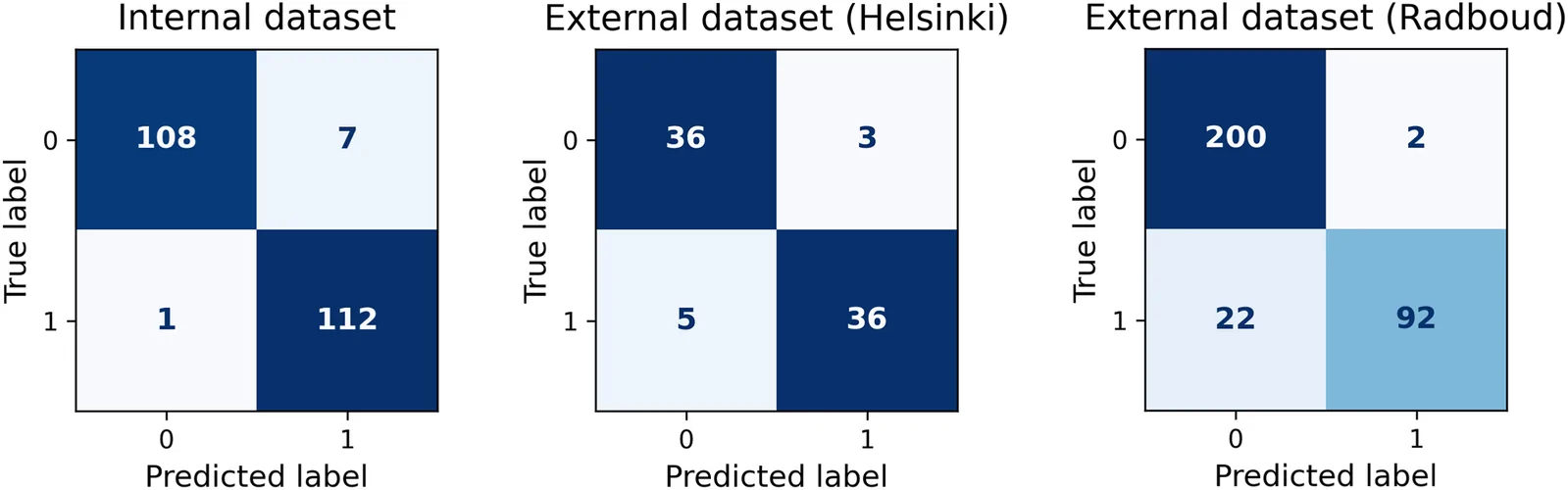
Model confusion matrices.

**Fig 4:**
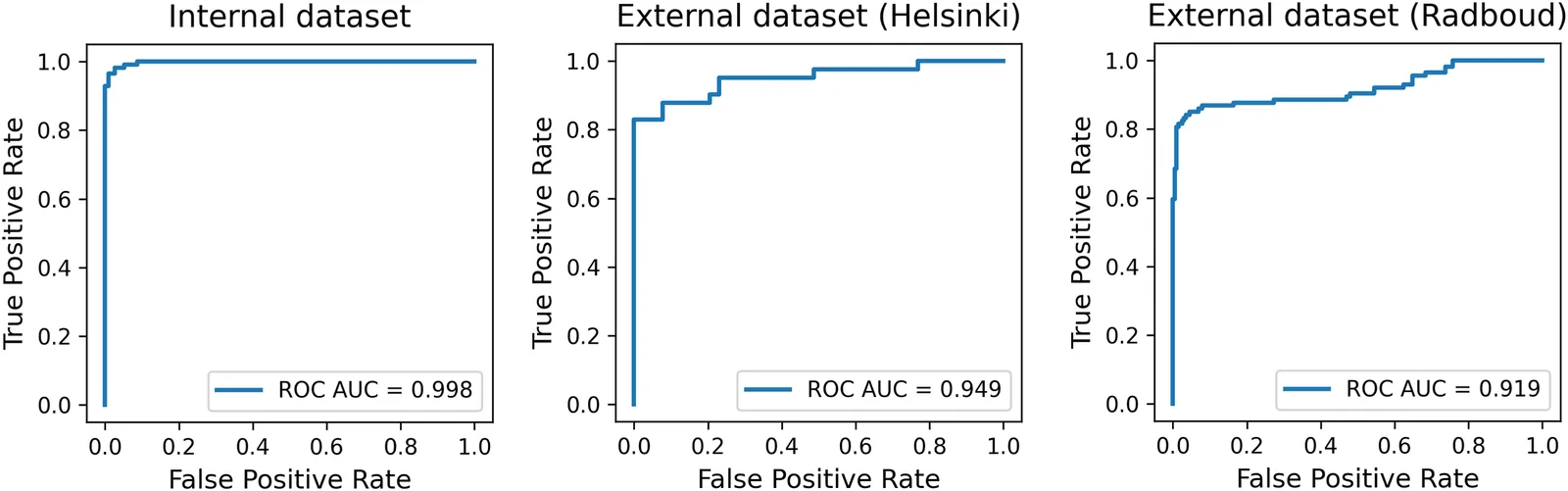
Model ROC curves. *AUC*, Area under the receiver operating characteristic curve; *IoU*, Intersection-over-Union; *ROC*, receiver operating characteristic.

A summary of model performance across the internal and external data sets is presented in Table II. On the internal test set, the model achieved a slide-level accuracy of 97.0% and an AUC of 0.998. The corresponding receiver operating characteristic curve and confusion matrix are shown in Figs 3 and 4, respectively.

**Table II:** Summary of model performance on the internal data set test set and the external data sets, Wilson 95% CIs is used for all metrics except for IoU where *t*-distribution was used

	Internal data set test set	External data set (Helsinki)	External data set (Radboud)
Sample size	228	80	316
Precision	0.941 (0.883-0.971)	0.923 (0.797-0.973)	0.978 (0.926-0.994)
Recall (sensitivity)	0.991 (0.951-0.998)	0.878 (0.745-0.947)	0.807 (0.725-0.869)
Specificity	0.939 (0.881-0.970)	0.923 (0.797-0.973)	0.990 (0.965-0.997)
Accuracy	0.965 (0.932-0.982)	0.900 (0.815-0.948)	0.924 (0.889-0.948)
AUC	0.998	0.949	0.919
IoU	0.41 ± 0.046 [Dice 0.58 ± 0.046]	-	-

*AUC*, Area under the receiver operating characteristic curve; *CIs*, confidence intervals; *IoU*, Intersection-over-Union.

### External validation on slide level

On the external data set from Helsinki University Hospital, the model achieved an accuracy of 90.0% with an AUC of 0.95. Performance on the external data set from Radboud University Medical Center yielded an accuracy of 92.0% and an AUC of 0.92. The receiver operating characteristic curves and confusion matrices for both external cohorts are shown in Figs 3 and 4, respectively.

### Pixel-wise validation on internal data

**Fig 5:**
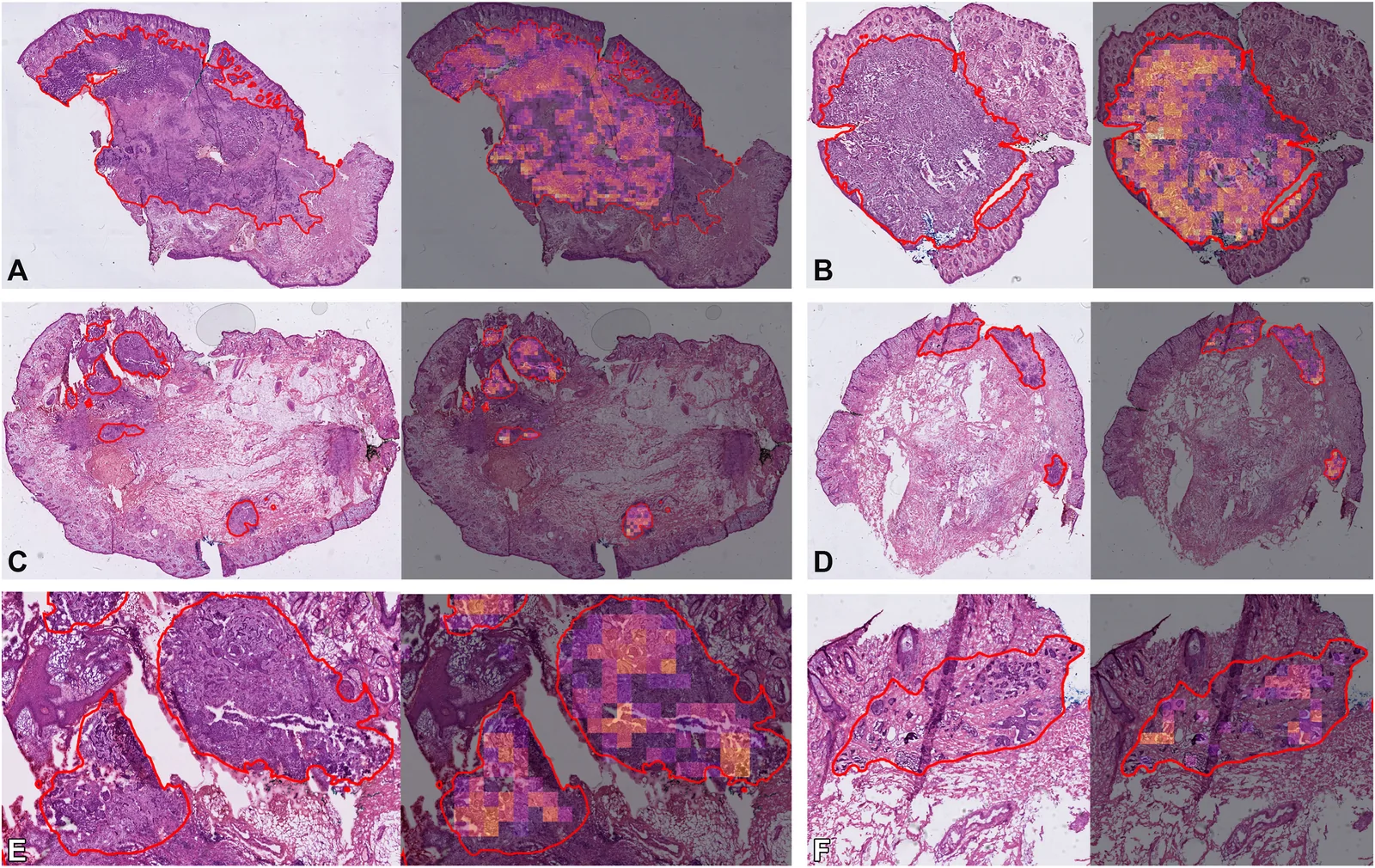
Image pairs of whole-slide images displaying tumor region annotations alone (*left*) combined with heatmaps (*right*). The annotated points were used to calculate IoU. IoU is **(A)** = 0.73 (Dice 0.84), **(B)** = 0.85 (Dice 0.92), **(C)** = 0.63 (Dice 0.77), and **(D)** = 0.54 (Dice 0.70); **(E)** = zoomed in area of **(C)**; **(F)** = zoomed in area of **(D)**. Hematoxylin and eosin stain. *IoU*, Intersection-over-Union.

The model achieved an IoU score of 0.41 ± 0.046 (Dice 0.58 ± 0.046) on the internal data set test set, reflecting overlap between predicted regions derived from heatmaps and the ground-truth annotations. Examples of the heatmaps from the model compared to the annotations are shown in Fig 5.

**Fig 6:**
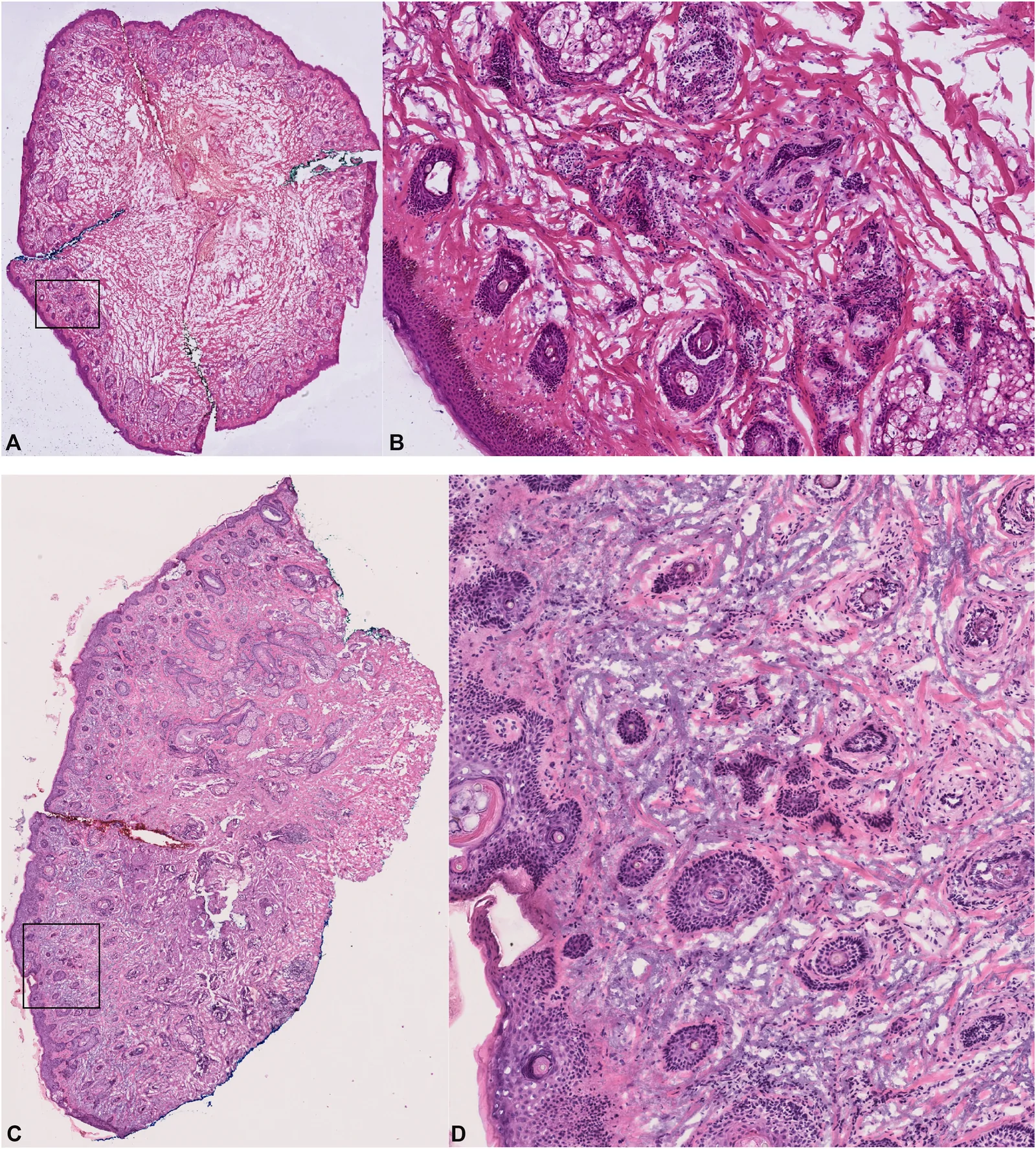
Examples of challenging, misclassified cases from the internal data set: **(A)** A case showing a small BCC focus that was originally misclassified as nontumor by pathologists but correctly identified by the AI model. **B,** A zoomed-in view of the same BCC focus. **C,** A false-positive case in which the AI model misclassified a proliferating hair follicle as BCC. **D,** A zoomed-in view of the hair follicle structure. Hematoxylin and eosin stain. *AI*, Artificial intelligence; *BCC*, basal cell carcinoma.

Examples of falsely classified cases in the internal data set are shown in Fig 6.

## Discussion

This study demonstrates that a weakly supervised MIL approach using a pathology foundation model can achieve highly accurate slide-level BCC detection on MMS frozen sections without requiring pixel-level labels. The model reached near-perfect internal test performance (accuracy 97.0%, AUC: 0.998) and retained strong performance on independent external data sets (accuracies >90%). Furthermore, heatmaps visualized diagnostically relevant regions, enhancing model interpretability.

Previously, Zon et al applied a supervised learning approach based on segmentation in binary classification of 171 MMS slides (of which 73 contained BCC and were manually annotated at the pixel level) as positive or negative for tumor presence and achieved an AUC of 0.90.^13^ However, when applied to more extensive data sets, this approach of manual annotation is time-consuming to perform and less scalable than, for example, the approach used by Campanella et al who used weakly supervised MIL to handle large data sets efficiently with sensitivity and specificity of up to 94% and an AUC of 0.99.^14^ However, a limitation in their study was the lack of heatmaps. Our study reached similar results with more limited data, possibly as a benefit of using a pretrained pathology foundation model and produced heatmaps. In another study, Bonefille et al applied a supervised segmentation approach to localize specific BCC foci, achieving an AUC of 0.99 and a sensitivity of 96% for BCC detection.^15^ Varra et al used a similar approach in their study, but only reached a sensitivity of 71%, likely due to scaling down the resolution of the images due to limited processing power available for the algorithm.^16^ A recent study by Geijs et al used AI with weakly supervised learning for detection of BCC in MMS slides reaching an AUC of 0.96 for slide-level performance and an AUC of 0.93 when applying the algorithm to an independent external data set. However, the accuracy of the resulting attention maps for localizing tumor regions was limited, with notable false-positive findings. This reduced localization precision may risk increasing, rather than decreasing, the time required for slide evaluation during MMS.^17^

From a technical perspective, our results using a weakly supervised framework are notable because MMS slides exhibit large intraslide heterogeneity, and the positive signal may occupy only a very small fraction of the tissue area. Consequently, methods relying on predefined minimum lesion-size thresholds, used in several prior histopathology pipelines to reduce noise, are not appropriate in this setting. Small tumors foci may occupy only a minute fraction of the tissue area, requiring detectors to remain sensitive even when the positive content is sparse. Our results show that our model can learn to detect these rare instances and still produce interpretable attention maps for model inspection.

To quantify spatial alignment between predicted attention and annotated tumor regions, we evaluated localization using the IoU metric and obtained a mean IoU of 0.41 ± 0.046 (Dice 0.58 ± 0.046). IoU is a strict overlap-based metric that penalizes even small boundary deviations, partial coverage, fragmented detections, or slight spatial shifts between prediction and annotation. This makes IoU substantially harsher than many localization evaluations reported in weakly supervised MMS pathology literature. For example, Geijs et al^17^ assessed attention-map localization primarily using sensitivity (ie, whether tumor regions are detected) combined with the number of revision false positives per slide, reporting attention map sensitivity of 0.853 without filtering (≈8 revision false positives per slide) and 0.873 after removing detections smaller than 200 μm (≈2 false positives per slide). Importantly, these metrics do not require tight spatial overlap with full tumor contours; rather, they measure whether tumor is flagged somewhere and how many false detections are produced. In contrast, IoU requires close correspondence of predicted and annotated tumor extent and is therefore more conservative. Consequently, while an average IoU of 0.41 ± 0.046 (Dice 0.58 ± 0.046) only suggests a moderate spatial overlap, the results could be interpreted as meaningful localization performance given the strictness of the metric. Thus, attention maps’ ability to highlight relevant areas for tumor detection may offer insights into the model’s focus areas.

The moderate IoU and Dice values likely reflect differences in annotation strategy, particularly with respect to tumor boundary delineation and whether surrounding tumor stroma was included (Fig 1), resulting in reduced agreement between predicted and reference regions despite accurate tumor localization (Fig 5). More standardized annotation criteria, particularly regarding stromal inclusion, may have improved spatial overlap metrics and heatmap performance. Furthermore, since the tumor regions were not annotated in the external data set images, no external localization validation using IoU calculations was available, which is a clear limitation of the study.

A detailed review of the originally falsely classified cases in both the internal and external data sets identified 1 incorrectly labeled case (false negative) in the internal data set (Fig 6, *A* and *B*) and 4 incorrectly labeled cases in the external Radboud data set (3 false positives and 1 false negative) by the pathologists. In these cases, the model prediction was correct, and the original ground-truth diagnoses were revised. This underscores the critical importance of an accurate ground truth when assessing diagnostic AI systems and the challenges associated with establishing a reliable reference standard for AI pathology studies.

Review of the remaining cases that were incorrectly classified by the AI model revealed that the only false-negative case in the internal data set was characterized by a very low tumor burden. False-positive cases were most commonly associated with benign epithelial proliferations, proliferating actinic keratosis bundles, hair follicle structures, inflammation, and stromal changes that histologically resembled BCC (Fig 6, *C* and *D*). Furthermore, tissue folds were present on the misclassified slides.

The false-negative cases in the Helsinki data set were associated with a very small tumor burden, often located within scar tissue or tissue folds. Many of these lesions were difficult to identify even on expert review because of their limited extent and their presence within areas of inflammation, scar tissue, or actinic damage. In several false-positive cases, technical factors such as tissue folds, tangential sectioning, focal blurring, or incomplete tissue representation further complicated detection.

Expert dermatopathology review of the false-negative cases from the Radboud cohort revealed that 6 of the 22 cases contained only very small tumor foci, whereas the remaining 16 cases showed clearly detectable tumor. The relatively high number of false-negative cases may be attributable to differences in scanner technology, tissue sectioning procedures, and the use of older glass slides. Nevertheless, expert review indicated that the slides were generally well preserved. Among the false-positive cases, 1 was explained by the presence of a detached tumor fragment located outside the main tissue section, which was identified by the AI model as tumor. The second false-positive case was associated with tissue folds that likely contributed to the misclassification.

False negatives behaved differently between the 2 external data sets and represent the primary source of performance variation under external validation. This is reflected in the sensitivity/recall decreasing from 0.878 in the Helsinki data set to 0.807 in the Radboud data set despite both data sets maintaining overall accuracies >90%. Technically, this pattern is consistent with domain shift affecting the separability of positive instances and underscores the risk of clinically significant false negatives. Sensitivity in the external data sets could potentially be improved by augmenting the fine-tuning data set with external cases, as training was performed exclusively on internal data.

The average processing time for a single MMS slide was 47.2 seconds including generating heatmaps. While further optimization may be possible, this runtime suggests that the algorithm could be feasibly integrated into the MMS workflow without causing substantial delays.

From a clinical perspective, accurate attention maps may enhance intraoperative workflow by directing focus to regions likely to contain BCC, potentially improving diagnostic accuracy and reducing oversights. Thus, AI-assisted analysis of MMS frozen sections has the potential to serve as a decision support tool during routine surgery. However, prospective studies are needed to evaluate its performance, safety, and impact on clinical workflow before clinical implementation can be considered.

## Conclusions

Although the success of MMS currently relies on the expert-dependent interpretation of frozen sections, this study addresses the need for efficiency by developing an MIL model for automated BCC detection. Internal validation revealed high diagnostic performance, while external testing confirmed robust generalizability with high accuracies. Furthermore, heatmap visualizations provided interpretable insights into the model’s decision-making regarding tumor localization. These results demonstrate that a weakly supervised approach showed promising retrospective performance as a decision support tool and may provide reliable, transparent intraoperative diagnostic support during MMS. However, prospective workflow validation is required before clinical implementation.

### Availability of data and materials

The data sets generated and/or analyzed during the present study will be available at the BCCC data set https://doi.org/10.23698/aida/bccc.

### Declaration of generative AI and AI-assisted technologies in the writing process

During the preparation of this work the authors used generative AI tools for language editing and readability improvement. After using this tool/service, the authors reviewed and edited the content as needed and take full responsibility for the content of the publication.

## Conflicts of interest

None disclosed.
